# Severe gastric distension in a teenage boy

**DOI:** 10.1002/jpr3.70266

**Published:** 2026-11-12

**Authors:** Erica Chang, Sharon Wolfson, Robert O. Heuckeroth, Livija Medne, Tomoki T. Nomakuchi, Jill Murrell, Sara L. Reichert, Brooke Weckselblatt, Jennifer Webster

**Affiliations:** ^1^ Department of Pediatrics Division of Pediatric Gastroenterology, Children's Hospital of Philadelphia Philadelphia Pennsylvania USA; ^2^ Perelman School of Medicine at the University of Pennsylvania Philadelphia Pennsylvania USA; ^3^ Department of Pediatrics Division of Human Genetics, Children's Hospital of Philadelphia Philadelphia Pennsylvania USA; ^4^ Genomic Diagnostic Laboratory, Division of Genomic Diagnostics, Children's Hospital of Philadelphia Philadelphia Pennsylvania USA

**Keywords:** gastric dilation, MYH11, variant of uncertain significance, visceral myopathy

## Abstract

A 16‐year‐old male presented with acute abdominal pain, nausea, and distension and was found to have severe gastric dilation on imaging. Antroduodenal manometry showed erythromycin‐induced phase 3 migrating motor complex beginning in the antrum but post‐prandial antral hypoactivity. Mitochondrial DNA sequencing was normal. Exome analysis revealed a rare variant of uncertain significance in the smooth muscle myosin heavy chain 11 (*MYH11*) gene (NM_002474.3: c.4123G>A, p.Asp1375Asn), which is necessary for smooth muscle contraction. Pathogenic *MYH11* alterations cause multiple disorders, including autosomal dominant (AD) familial thoracic aortic aneurysm, AD visceral myopathy, and autosomal recessive megacystis‐microcolon‐intestinal‐hypoperistalsis syndrome. Amino acid p.Asp1375 is within the rod region of the MYH11 protein that self‐assembles to form thick filaments; thus, pathogenic variants in this region may affect filament formation and impair interactions with thin filaments. While the phenotype overlap is compelling, further research is needed to determine the clinical significance of this alteration.

## 1 INTRODUCTION

Myosin heavy chain 11 (*MYH11)* encodes the *MYH11* protein which is a core component of smooth muscle thick filaments. Thick filaments interact with thin filaments to cause smooth muscle contraction. In previous studies, pathogenic variants in the *MYH11* gene have been associated with autosomal dominant (AD) familial thoracic aortic dissection, AD visceral myopathy, and autosomal recessive megacystis‐microcolon‐intestinal hypoperistalsis syndrome (MMIHS).^1^ Here, we describe a teenage male with severe acute gastric distension who was found to have a rare variant of uncertain significance (VOUS) in *MYH11* (NM_002474.3: c.4123G>A, p.Asp1375Asn), which is hypothesized to underlie his clinical presentation.

## 2 CASE REPORT

A 16‐year‐old male without significant gastrointestinal (GI) history presented with days of progressive nausea, abdominal pain, and distension after eating a cheesesteak sandwich. He had two similar episodes within the past year that resolved with bismuth subsalicylate and dimenhydrinate. Computed tomography (CT) showed severe gastric dilation (Figure 1) and dilation of the esophagus and duodenum with abrupt tapering at the third portion. While this finding is typically seen in superior mesenteric artery (SMA) syndrome, his subsequent abdominal ultrasound with Doppler revealed a normal aortomesenteric angle (between 25 and 60°) and distance (8.4 mm) making SMA syndrome unlikely. Additionally, his history did not fit the typical presentation of SMA syndrome with lack of notable weight loss; however, his body mass index was low (18.48 kg/m^2^, *z* score −1.03) in the setting of his tall stature (186.7 cm, 96%ile) relative to his weight (64.4 kg, 55.7%ile).

**Figure 1:**
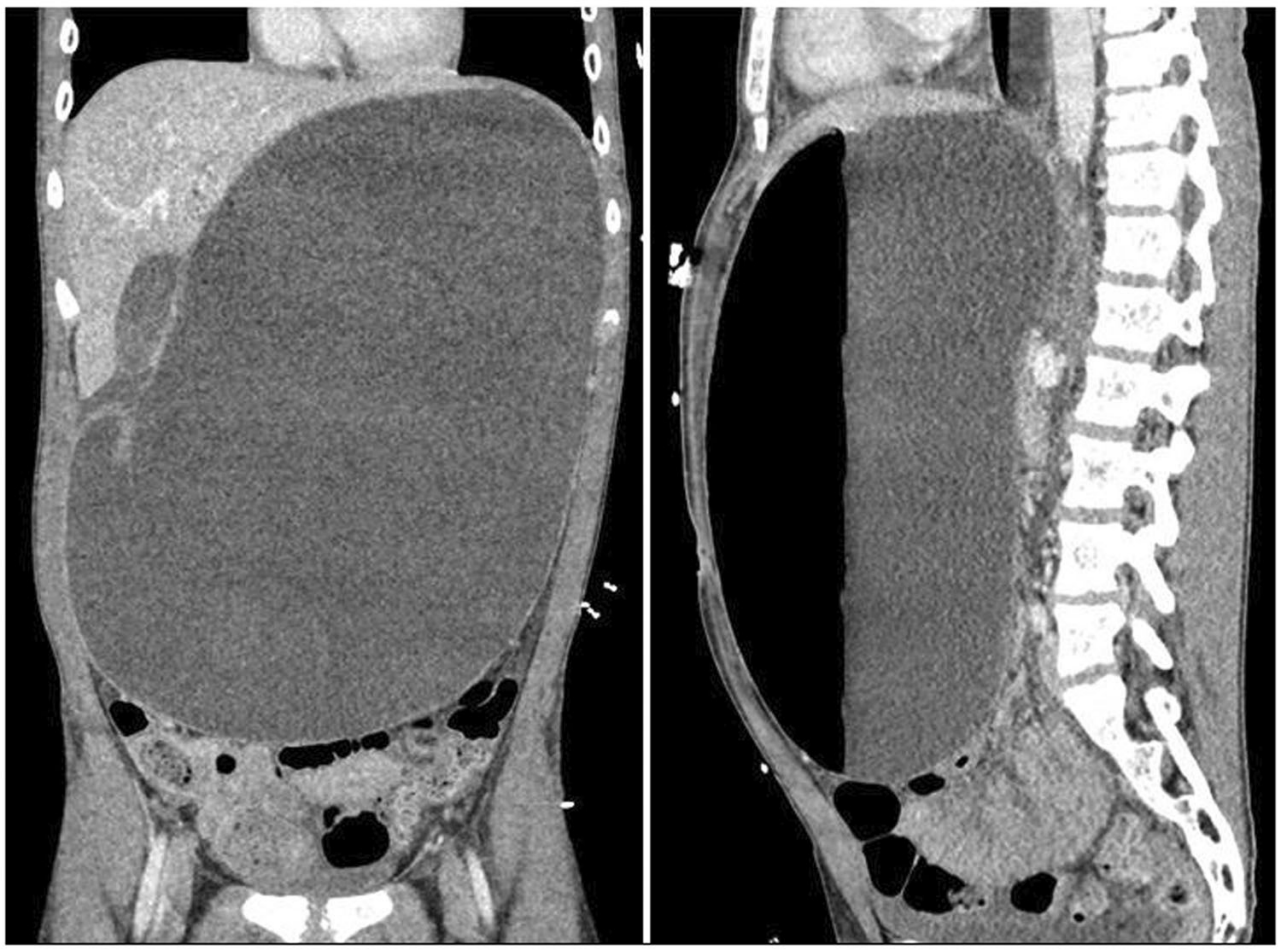
CT abdomen/pelvis at initial presentation demonstrated significant gastric distension (left coronal, right sagittal). CT, computed tomography.

A Salem sump was placed with significant initial output (>5 L). Bowel rest and a proton–pump inhibitor (PPI) were started. Repeat CT 2 days after presentation showed proximal duodenal decompression, nondistended third portion of the duodenum, and decreased dilation of the distal esophagus and stomach. Esophagogastroduodenoscopy (EGD) revealed deep ulcerations within the gastric body and edematous/erythematous gastric mucosa (Figure 2). Pathology showed neutrophilic infiltrate in the esophagus, antrum, and gastric body, with minimal/focal active inflammation in the duodenum. No organisms or viral cytopathic effects were identified. Nasojejunal (NJ) feeds were initiated and tolerated. Upper GI demonstrated ready transit of contrast into the jejunum.

**Figure 2:**
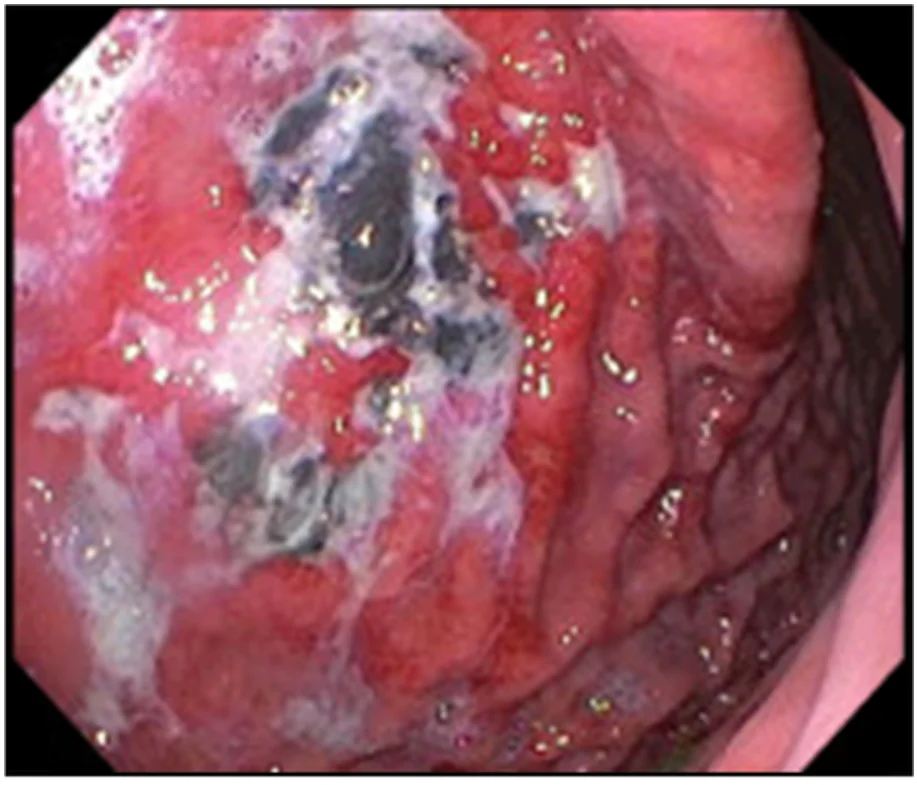
EGD revealed deep ulcerations and erythema within the gastric body. EGD, esophagogastroduodenoscopy.

Antroduodenal manometry (ADM) showed a normal response to erythromycin with a phase 3 migrating motor complex originating in the gastric antrum with anterograde propagation down >30 cm of small bowel. Meal challenge revealed antral hypoactivity with only contractions during the first 15 min of the meal and none beyond (Figure 3). Erythromycin was started based on ADM results, and he was able to tolerate pureed foods with removal of sump. By hospital day 15, he tolerated a regular diet and was discharged on day 18 on oral/NJ feeds, lansoprazole, and erythromycin.

**Figure 3:**
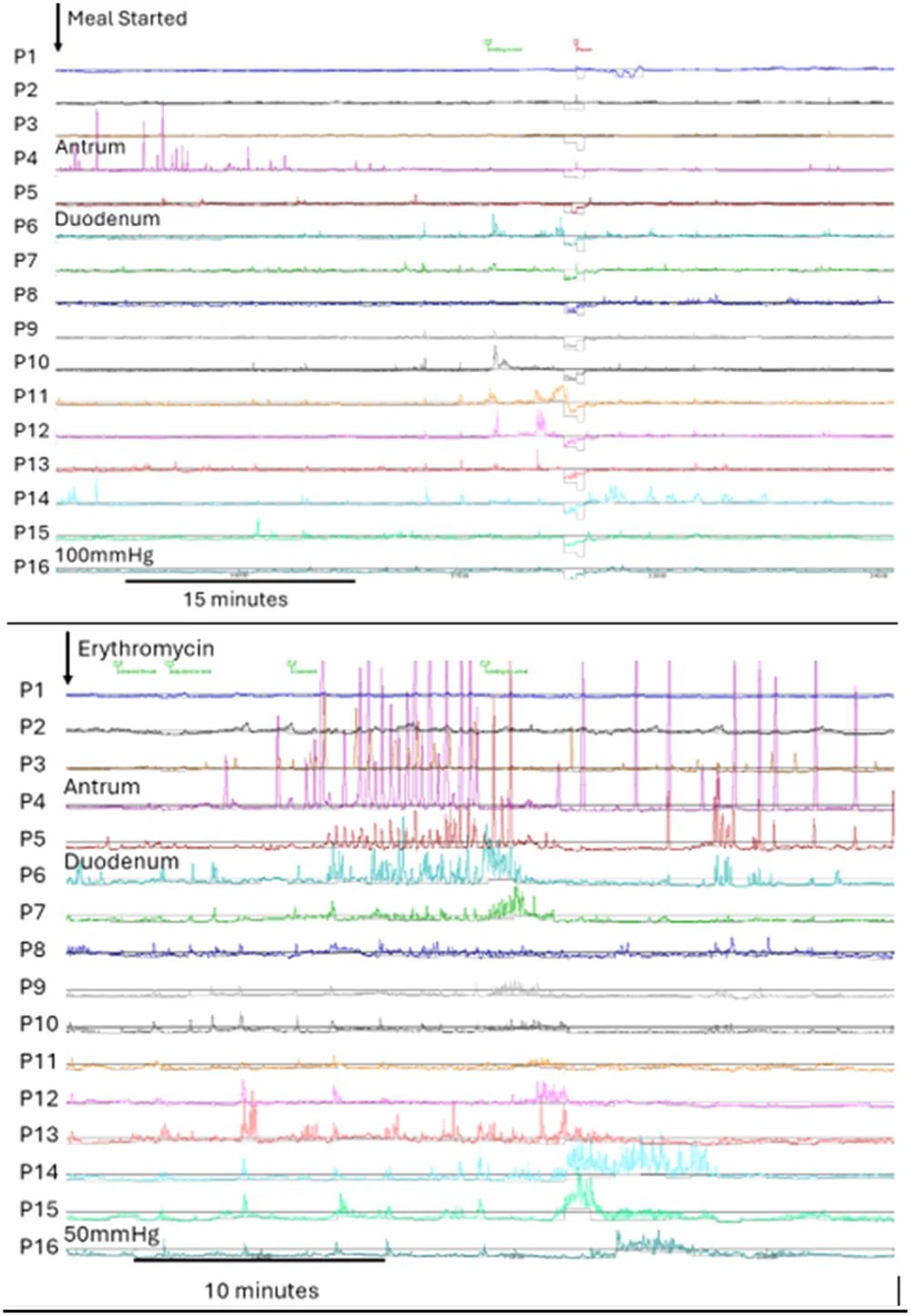
Antroduodenal manometry showed antral hypoactivity with a phase 3 migrating motor complex starting in the antrum in response to erythromycin.

Approximately 1 month after discharge, the NJ tube was removed with normal oral diet. Repeat endoscopy 4 months after the initial EGD showed healed ulcers and focal active gastritis. He was continued on PPI, but erythromycin was stopped. Abdominal X‐ray (AXR) a month later showed a mildly distended stomach, and a gastric emptying scan a couple weeks afterwards was normal. Mitochondrial DNA sequencing was normal, and exome analysis performed from short‐read genome sequencing data revealed a paternally inherited VOUS in the *MYH11* gene (NM_002474.3: c.4123G>A, p.Asp1375Asn). There was no family history of *MYH11*‐related phenotypes.

He remained well until 20 months after initial presentation when he developed similar symptoms during vacation requiring admission and sump decompression. After returning home, he presented to our ED where AXR redemonstrated severe gastric distension. CT abdomen with angiography (CTA) showed significant dilation of the first and second portions of the duodenum with abrupt narrowing of the third portion. The aortomesenteric angle (20°) and distance (5 mm) were abnormally decreased. This suggested SMA syndrome in the setting of weight loss from 66.1 kg 3 months prior to 60.7 kg (after decompression). As a result, nutritional rehabilitation was recommended, and he was discharged on clears/NJ feeds with plans for gastrojejunostomy tube placement. Repeat CTA after weight restoration revealed resolution of SMA compression, indicating mechanical obstruction related to weight loss and external compression from the dilated stomach.

## 3 DISCUSSION

Visceral myopathy syndromes are commonly caused by pathogenic variants in genes essential for smooth muscle contractility, including *MYH11, ACTG2, FLNA, MYLK, MYL9, LMOD1*, and *ACTA2*.^2^ The *MYH11* missense variant found in this case is present in gnomAD v4 in 30/1,613,670 alleles in a heterozygous state and reported in ClinVar as a VOUS in six individuals, five of whom had aortic aneurysm as clinical indication for testing.^1^ To date, pathogenic alterations in *MYH11* associated with AD visceral myopathy have included frameshift variants in the last coding exon of the gene affecting the SM2 isoform, resulting in protein elongation.^3^, ^4^, ^5^ Based on the Human Gene Mutation Database, dominant‐negative acting predominantly missense variants in *MYH11* have been found in patients with familial thoracic aortic aneurysm and related aneurysm phenotypes.^6^ Loss‐of‐function alleles are typically recessive with a second pathogenic variant affecting the other allele. For example, a 2019 case report described a MMIHS phenotype in a girl with compound heterozygous variants of the *MYH11* gene: a paternally inherited c.379C>T, p.Pro127Ser missense variant and a maternally inherited 16p.13.11 microdeletion encompassing the *MYH11* gene.^7^

Although this specific p.Asp1375Asn (a.k.a. D1375N) variant does not have an established association with visceral myopathy, it may contribute to smooth muscle dysfunction since the MYH11 protein is a major component of thick filaments. In this variant, aspartic acid with a negatively charged carboxylic acid group is converted into a neutral asparagine residue within the myosin rod domain. This domain allows for dimerization and polymerization to form thick filaments. Thus, variants could theoretically compromise thick filament assembly, alter structure, or reduce stability, potentially impairing interactions with thin filaments. Additionally, actin‐myosin interactions resist passive stretch. When these interactions are impaired, the stomach may be predisposed to stretching after a large meal. Given the Law of Laplace (greater force is needed to contract larger tubes), it may be possible that a large meal could have distended this teenager's stomach to the point where the force required for contraction exceeded the force that could be generated.

In support of our hypothesis that the variant could cause smooth muscle weakness, other AD pathogenic *MYH11* variants cause pediatric intestinal pseudo‐obstruction (PIPO).^3^, ^4^, ^5^, ^8^ PIPO mimics mechanical obstruction despite the absence of luminal blockage. While our patient does not fully meet European Society for Paediatric Gastroenterology, Hepatology and Nutrition criteria for PIPO, his symptoms are similar to the expected presentation with abdominal distention, vomiting, and abdominal pain.^8^, ^9^ Given his asymptomatic periods, this variant may cause a relatively mild PIPO phenotype that allows for adequate force generation in the absence of significant luminal distention, inflammation, or other stressors.^10^

It is possible this patient experienced transient gastric dysmotility unrelated to *MYH11*‐related visceral myopathy as his symptoms were short‐lived (albeit recurrent) and primarily limited to the stomach. Additionally, he did not have signs of chronic dysmotility or smooth muscle dysfunction beyond the GI tract (such as urinary retention, bladder dilation, or dysphagia). While colonic manometry could provide further evidence of smooth muscle dysfunction by differentiating myopathic from neuropathic processes, we did not pursue testing as the benefits did not outweigh potential risks in the absence of significant lower GI symptoms (only had intermittent constipation). In addition to SMA syndrome, other differentials include acute gastric atony or transient functional obstruction; however, there were no risk factors, such as chronic or severe systemic illness, neurologic events, prior surgeries, infections, or medications (i.e., anticholinergics, opiates, etc.). Further studies are needed to elucidate the biochemical and clinical impact of this variant.

## 4 CONCLUSION

While the *MYH11* (NM_002474.3: c.4123G>A, p.Asp1375Asn) variant has not been reported to be pathogenic to date, it may cause smooth muscle dysfunction by altering thick filament assembly and structure, thereby impairing cross‐bridge cycling and resistance to passive stretch. This may lead to a mild PIPO phenotype, but further research is needed to determine the significance of this variant.

## CONFLICTS OF INTEREST STATEMENT

Robert O. Heuckeroth is a member of the Scientific Advisory Boards for the World Visceral Myopathy Foundation and for Neurenati Therapeutics. The remaining authors declare no conflicts of interest.

## ETHICS STATEMENT

Informed parental consent/patient assent was obtained for publication of case details.
